# Premier Impressed

**Published:** 1920-02-28

**Authors:** 


### Premier Impressed.

### Action Forshadowed to Check High Prices.

The widespread uneasiness about the prices of food and other commodities, together with the agitation on the subject throughout the country, seems at last to have made an impression upon the Prime Minister, for the *Daily Chronicle*, which is supposed to be representative of the Prime Minister's points of view, says it understands new measures to deal with the pressing problem of high prices are being considered by the Cabinet.

The efficacy of the policy of decontrol, which has been pursued since the Armistice, is now being questioned in certain quarters, and suggestions have been made to the Government that fresh methods are required in order to protect the interests of the consumer.

It is now regarded as practically certain that the Ministry will not be closed down, and, in view of the recent reports of the committees under the Profiteering Act, representations are being made to the Government that some measure of control at any rate is necessary, not only in the case of food, but also in such commodities as wool, leather, and cotton.

The consumer has had to make considerable commotion before his circumstances have received attention, and it looks as though even now political expediency, rather than the existing conditions, is the driving force.

It is pointed out that the Ministry of Food, which is not represented in the Cabinet, is the only Government department which stands for the interests of the consumer, and also that the question of food prices is only a small part of the problem of reducing the cost of living.

The Prime Minister is believed to be considering the proposal to invest the Food Ministry with wide powers in respect to fixing prices.

Mr. McCurdy, the Chairman of the Central Profiteering Committee, is known to hold the view that new forms of control will have to be evolved with the active co-operation of the trades concerned.

His belief is that once the Government adopts a strong policy, the representatives of the various industries will be quite willing voluntarily to limit their own profits, and that it will only be necessary to keep the powers invested by the Profiteering Act in the background.

The *Chronicle* says, at present the chief difficulty in the way of a systematic policy for lowering prices lies in the fact that the divided authority of the Board of Trade, the Treasury, and the Food Ministry hampers effective action.

The standpoint of the advocates of recontrol is that the decision on such matters of these two departments cannot be helpful to the interests of the consumer, and that their powers should be transferred to the Food Ministry, which would, in effect, assume the functions of a Ministry for the Consumer.

It is rather late in the day to put ineffective action down to muddled departmental administration, and with the fatuous failure of the profiteering regulations in mind, most people will prefer to wait and see what these inspired assurances really mean. They will not be over-hasty to count the chickens before they are hatched, for even when they are hatched they may still be prohibitive in price. The public requirements can be stated in perfectly simple terms—namely, that effectual measures be taken to prevent exploitation of present needs. The only test of Government action is whether it definitely and patently secures these requirements. It is futile to point to Acts of Parliament designed to attain these ends. The acts of Parliament and of the departments must attain those ends.

